# GtrS and GltR form a two-component system: the central role of 2-ketogluconate in the expression of exotoxin A and glucose catabolic enzymes in *Pseudomonas aeruginosa*

**DOI:** 10.1093/nar/gku496

**Published:** 2014-06-11

**Authors:** Abdelali Daddaoua, Carlos Molina-Santiago, Jesús de la Torre, Tino Krell, Juan-Luis Ramos

**Affiliations:** Department of Environmental Protection, CSIC-EEZ, C/Profesor Albareda 1, E-18008 Granada, Spain

## Abstract

In the human pathogen *Pseudomonas aeruginosa*, the GltR regulator is required for glucose transport, whereas GtrS is a sensor kinase that plays a key role in mediating bacteria–host interaction and pathogen dissemination in the host. We show that GtrS and GltR form a two-component system that regulates the expression from the promoters P*_edd/gap-1_*, P*_oprB_* and P*_glk_*, which control the expression of genes involved in glucose metabolism and transport. In addition, the GtrS/GltR pair regulates the expression of *toxA* that encodes exotoxin A, the primary virulence factor. Microcalorimetry-based ligand screening of the recombinant GtrS ligand-binding domain revealed specific binding of 2-ketogluconate (2-KG) (*K*_D_ = 5 μM) and 6-phosphogluconate (*K*_D_ = 98 μM). These effectors accelerate GtrS autophosphorylation, with concomitant transphosphorylation of GltR leading to a three-fold increase in transcription. Surprisingly, *in vivo* a similar increase in expression from the above promoters was observed for the mutant deficient in GltR regardless of the presence of effectors. The GltR operator site was found to contain the consensus sequence 5′-tgGTTTTTc-3′. We propose that 2-KG is a key metabolite in the stringent transcriptional control of genes involved in virulence and glucose metabolism. We show that GltR is a transcriptional repressor that is released from DNA upon phosphorylation.

## INTRODUCTION

The ubiquitous Gram-negative bacterium *Pseudomonas aeruginosa* is an opportunistic human pathogen, which is a frequent cause of hospital-acquired infections, including ventilator-associated pneumonia and catheter infections in immunocompromised patients (1). Furthermore, *P. aeruginosa* is an etiologic agent of ear (2) and urinary tract infections (3) and causes infections in severely burned individuals (4) as well as in patients who suffer from cystic fibrosis (5). The establishment of *P. aeruginosa* infection is accompanied by the synthesis of several extracellular and cell-associated virulence factors, among which is exotoxin A, encoded by the *toxA* gene (6,7). Similar to other extracellular virulence factors such as diphtheria-, cholera- and pertussis-toxin, exotoxin A is an ADP-ribosyl transferase that modifies host elongation factor-2, leading to a reduction of protein synthesis and eventually cell death (7).

**Figure 1.**
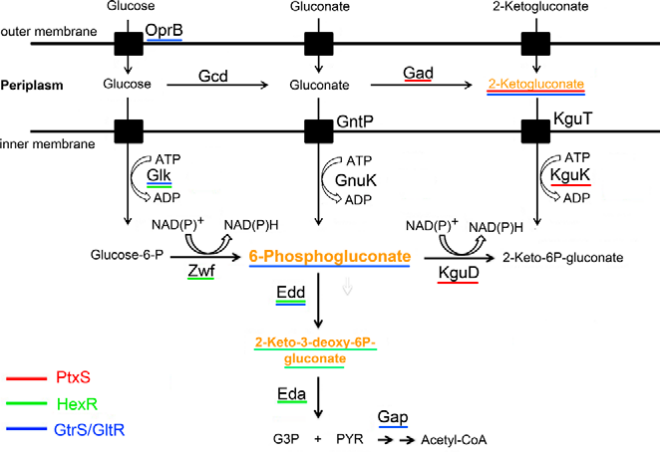
Schematic representation of the glucose metabolism in *Pseudomonas* as deduced from gene annotations and functional analysis. Proteins underlined in different colors indicate the transcriptional regulators that participate in their transcriptional regulation. Effector molecules are shown in yellow and are underlined according to the regulator system they bind to. Gcd, glucose dehydrogenase; Gad, gluconate dehydrogenase; KguD, 2-KG reductase; Glk, glucokinase; GnuK, gluconokinase; KguK, 2-KG kinase; Zwf-1, glucose-6-phosphate 1-dehydrogenase; Pgl, 6-phosphoglucose lactonase; Edd, phosphogluconate dehydratase; Eda, 2-keto-3-deoxy gluconate aldolase; GntP, gluconate permease; KguT, Gap, glyceraldehyde-3-phosphate dehydrogenase; 2-KG transporter; PYR, pyruvate.

Previous work in our laboratory has shown that the regulation of expression of glucose catabolic genes and that of *toxA* are tightly linked (8). In bacteria of the genus *Pseudomonas*, glucose can be metabolized by up to three different routes that converge into 6-phosphogluconate (6PG) (Figure 1). Glucose in the periplasm is oxidized to gluconate, which can be transported to the cytoplasm and phosphorylated to 6PG by gluconate kinase or can be oxidized further in the periplasm to 2-ketogluconate (2-KG), which enters the cytoplasm and is converted into 6PG via 2-keto-6-phosphogluconate; these two reactions are carried out by the KguK and KguD enzymes. Further metabolism of 6PG yields G3P and pyruvate and the latter reaction performed by the glyceraldehyde-3-phosphate dehydrogenase (Gap) yields acetyl-CoA (Figure 1). Studies in *P. putida* and *P. aeruginosa* have shown that the PtxS regulator controls its own expression at P*_ptxS_* as well as that of the operon formed by the *gad* genes at P*_gad_* and the *kguD* genes at P*_kgu_* (8,9,10,11). PtxS was found to bind exclusively 2-KG as an effector molecule and the binding of 2-KG to DNA-bound PtxS caused the protein to dissociate from the DNA, enabling efficient transcription (9). Previously, it was shown that in *P. aeruginosa*, PtxS in conjunction with PtxR is involved in the transcriptional control of *toxA* expression (12,13,14). The mechanism of PtxS/PtxR-mediated control of *toxA* expression has recently been revealed (8). PtxR was found to bind to the promoter P*_toxA_* and PtxS was identified to bind to the DNA-bound PtxR. The resulting heterocomplex inhibits transcription. However, the binding of 2-KG to PtxS leads to the dissociation of PtxS from the PtxR-DNA complex causing transcriptional activation (8). These data illustrate the intimate relationship between the transcriptional regulation of the expression of exotoxin A and glucose catabolic genes.

Another regulator in *Pseudomonas*, termed HexR, was also found to be involved in the regulation of glucose metabolism. The effector molecule recognized by HexR was identified to be another intermediate of glucose metabolism, namely 2-keto-3-deoxy-6-phosphogluconate (KDPG, Figure 1) (15). HexR controls the transcription from the promoters P*_zwf_*, P*_edd_* and P*_gap-1_* and regulates the expression of the Entner–Doudoroff enzymes 6PG dehydratase (Edd), 2-keto-3-deoxy-6-phosphogluconate aldolase (Eda), glucose-6-phosphate 1-dehydrogenase (Zwf) and glucokinase (Glk). HexR also controls expression of another transcriptional regulator, GltR (PA3192), which in turn controls expression of specific porins for the entry of glucose into the periplasmic space, as well as the *gtsABCD* operon for glucose transport through the inner membrane to the cytoplasm (16,17). Interestingly, the gene product of *gtrS* (PA3191), located adjacent to *gltR*, plays a key role during *P. aeruginosa*–host interactions and is required for optimal colonization and dissemination in a mouse model of infection (18). It was demonstrated that GtrS contributed to a modulation of the type III secretion system in response to host cells (18). However, the molecular basis for the action of GltR and GtrS is unknown. The analysis of GtrS and GltR sequences in InterPro suggests that GtrS has the functional domains of a sensor kinase, whereas GltR may be a response regulator protein. We show here that GtrS and GltR form a two-component system (TCS), which responds to 2-KG and 6PG. This novel TCS was found to regulate transcription of genes involved in glucose metabolism (*glk* and *edd*/*gap-1*) and transport (*oprB*) as well as *toxA* gene expression. Surprisingly, we found that the GltR response regulator is a repressor that is released from its target operators of the *toxA* and glucose metabolism genes upon phosphorylation. This system corresponds to another mode that guarantees a concerted regulation of carbohydrate metabolism and exotoxin A expression.

## MATERIALS AND METHODS

### Bacterial strains, plasmids and culture media

Strains and plasmids used in this study are listed in Table 1. Bacterial strains were grown in LB (Luria Bertania) medium or in modified M9 minimal medium (Na_2_HPO_4_: 6 g/l; KH_2_PO_4_: 3 g/l; NaCl: 0.5 g/l; NH_4_Cl: 1 g/l, 1-mM MgSO_4_, 0.3-mM CaCl_2_ and 0.2 ml/l of 1% ferric ammonium citrate) with 5-mM citrate as the sole C-source (19). When required, antibiotics were added to the culture medium to final concentrations of 25 μg/ml (kanamycin), 50 μg/ml (ampicillin) or 10 μg/ml (tetracycline). *Escherichia coli* strain DH5α was used for plasmid construction and *E. coli* BL21 (DE3) strain was used for protein overexpression.

**Table 1.** Strains and plasmids used in this study

Strains or plasmid	Genotype or relevant characteristics	Reference
**Strains**		
*P. aeruginosa* PAO1	Wild type, prototroph; Ap^r^	(a)
*P. aeruginosa* Δg*ltR*	*gltR*: pCHESIΩ-Gm;Gm^r^	This Work
*E. coli DH5αF’*	F’/*hsd*R17, *recA*1, *gyrA*	(a)
*E. coli* BL21 (DE3)	F^−^, *ompI*, *hsdS*_B_(r^−^_B_m^−^_B_) *gal*, *dam*, *met*	(a)
**Plasmids**		
pMBL-T	PCR product cloning vector, Ap^r^	Dominion
pET28a (+)	Km^r^, protein expression vector, x6His	Novagen
pMAL-C2	Maltose-binding protein in pMAL-C2 vector	Collection^a^
GltR pMBL	*gltR* gene in pMBL	This Work
GltS pMBL	*gltS* gene in pMBL	This Work
GtrS-Cter pMBL	*gtrS* cytosolic fragment in pMBL	This Work
GtrS-LBD pMBL	*gtrS* ligand-binding domain in pMBL	This Work
GltS-MBP pMBL	*GtrS-Maltose-Binding Protein* in pBML	This Work
GtrS-Cter pET28a	pET28a containing *gtrS* cytosolic fragment	This Work
GtrS-Lbd pET28a	pET28a containing *gtrS* ligand-binding domain	This Work
GltS pET28a	pET28a containing *gtrS* gene	This Work
GltR pET28a	pET28a containing *gltR* gene	This Work
GltR D56A pET28a	pET28a containing mutant D56A *gltR* gene	This Work
GltR-MBP pET28a	pET28a containing *gltR-Maltose-Binding Protein* gene	This Work
P*_oprB_*::pBML	*oprB* promoter in pMBL vector	This Work
P*_glk_*::pBML	*glk* promoter in pMBL vector	This Work
P*_edd/gap-1_*::pMBL	*edd/gap-1* promoters in pMBL vector	This Work
Bgal::*oprB*	pMP220 bearing the promoter region of the *oprB*, Tc^R^	This Work
Bgal::*kgl*	pMP220 bearing the promoter region of the *glk*, Tc^R^	This Work
Bgal::*edd*	pMP220 bearing the promoter region of the *edd*, Tc^R^	This Work
Bgal::*gap-1*	pMP220 bearing the promoter region of the *gap-1*, Tc^R^	This Work
Bgal::*toxA*	pMP220 bearing the promoter region of the *toxA*, Tc^R^	This work
pMBL::*gltR*::Gm	*gltR*::pCHESIΩ-Gm	This work

Tc^r^, Km^r^ and Ap^r^ stand for resistance to tetracycline, kanamycin and ampicillin, respectively.

^a^Collection of vectors and strains available at the Consejo Superior de Investigaciones Científicas, Granada, Spain.

### Transcriptional fusions to the ‘*lacZ* reporter gene

To obtain a transcriptional fusion of the promoter of the *toxA* gene and genes for carbohydrate metabolism to the ‘*lacZ* reporter gene, the corresponding promoter regions were amplified by polymerase chain reaction (PCR) using *P. aeruginosa* strain PA01 chromosomal DNA as a template and the primers specified in Supplementary Table S1. The resulting PCR products containing BglII and PstI restriction sites were cloned into plasmid pMBL-T (MolBiolab) (Table 1). The absence of mutations in the recombinant plasmids was verified by sequencing of the insert and flanking regions. The BglII-PstI fragments were subsequently excised from the pMBL-T derivatives and cloned into the pMP220 promoter trap-vector using the same restriction sites. The resulting plasmids were transformed into wild-type or mutant *P. aeruginosa* PA01.

### Generation of a *gltR* mutant

To generate the *gltR*::Gm mutant strain, a 394-bp DNA fragment covering the central part of the *gltR* gene was amplified by PCR from *P. aeruginosa* PA01 genomic DNA using primers ΔGltR.f and ΔGltR.r (Supplementary Table S1) and the resulting product was cloned into pMBL to yield pMBL::gltR. Subsequently, the resulting plasmid was digested with *BamHI* and *XhoI*, which liberated the *gltR* fragment. The plasmid pCHESI was also digested with *BamHI* and *Xho*I, to liberate the gentamicin resistance gene. The Gm resistance gene was ligated with the *gltR* fragment and the resulting chimeric DNA was cloned into pMBL digested with *BamHI* and *XhoI* to yield pMBL::*gltR*::Gm. This plasmid was electroporated into *P. aeruginosa* PA01 for homologous double recombination. Mutant strains were selected on Gm plates and the correctness of the mutation was verified by Southern blotting.

### β-Galactosidase assays

*Pseudomonas aeruginosa* PA01 and its isogenic *gltR* mutant were grown in minimal medium supplemented with 5-mM citrate and 10-μg/ml tetracycline. Cultures were grown in the presence and the absence of 5-mM 2-KG or 6PG. Overnight cultures were diluted to an OD_660_ of 0.01 in the same medium and growth was continued at 37°C for 3 h. Samples were taken and β-galactosidase activity was determined with *o*-nitrophenyl-β-d-galactoside as a substrate in permeabilized whole cells as previously described (20). At least three independent experiments were performed and the activity was expressed in Miller Units.

### Ribonucleic acid extraction and primer extension

*Pseudomonas aeruginosa* PA01 cells were grown in M9 minimal medium supplemented with 5 mM citrate as carbon source and 5 mM 2-KG as an effector. Ribonucleic acid (RNA) was extracted using the TRI reagent® protocol (Ambion) and the integrity of RNA was assessed by agarose gel electrophoresis. Primer extension reactions were performed as described by Marques *et al.* (21) using the primers indicated in Supplementary Table S1.

### Overexpression of recombinant proteins in *E. coli* and their purification

The following six recombinant proteins were expressed in *E. coli*: full-length GtrS and GltR, the ligand-binding domain of GtrS (residues 1–172, GtrS-LBD), the cytosolic fragment of GtrS (residues 269–465, GtrS-Cter), a GltR-maltose-binding protein fusion (GltR-MBP) and a GltR mutant in which D56 was replaced by A. For the construction of expression plasmids of the former four proteins, the corresponding DNA fragments were amplified by PCR using the primers listed in Supplementary Table S1. The resulting fragments were cloned into plasmid pMBL-T and the absence of mutations was verified by sequencing. Subsequently, the pMBL derivatives were digested with *Nde*I and *BamH*I, and the liberated inserts were cloned into pET28a(+) digested with the same enzymes. The resulting plasmids allowed the expression of the fusion protein with a His-tag at their C-terminus.

For the construction of the GltR-MBP fusion, plasmids GltR-pET28(a) and pMAL-C2 (containing the gene for the MBP) were digested with *Nco*I and *BamH*I and the resulting fragments were ligated. The recombinant DNA fragment was cloned into the pET28a(+) vector such that it expressed the recombinant protein bearing an MBP fusion at the N-terminus and a His-tag at the C-terminus.

To generate GltR D56A, a modified version of the Hemsley method (22) was used. The pair of partially overlapping mutagenic primers GltR D56Ar and GltR D56Af was used (Supplementary Table S1) to amplify the entire plasmid with a high-fidelity DNA polymerase, which generated nicked circular DNA. After PCR amplification with *Pfu Turbo* DNA polymerase (Agilent Technologies), the methylated template DNA was eliminated by digestion with *Dpn*I (an enzyme specific for methylated DNA). The resulting PCR products were electrotransformed into *E. coli* DH5α and colonies were selected on LB supplemented with kanamycim (50 μg/ml). Plasmid DNA from resulting clones was isolated and inserts and flanking regions were sequenced for verification.

For the overexpression of the six proteins, *E. coli* BL21 (DE3) containing the corresponding pET28(a) derivative was grown in 2-l conical flasks containing 250-ml LB supplemented with 25-μg/ml kanamycin. Cultures were incubated at 30°C with shaking and protein expression was induced by adding 0.5-mM isopropyl-β-d thiogalactopyranoside when the culture reached a turbidity of ∼0.6 at OD_660_. The cultures were then incubated overnight at 18°C prior to cell harvest by centrifugation at 20 000 x g for 30 min. Cells were then stored at −80°C.

Proteins GltR, GltR D56A, GtrS-LBD, GtrS-Cter and MBP-GltR are soluble proteins and were purified by affinity chromatography. Cells were suspended in 20-ml buffer A (50-mM Tris–HCl, pH 7.9, 300-mM NaCl, 1-mM dithiothreitol, 10-mM imidazole) containing a tablet of Complete^TM^ ethylenediaminetetraaceticacid (EDTA)-free protease inhibitor (Roche). Cells were broken by repeated French Press treatment at 1000 psi. The cell lysate was centrifuged at 20 000 x g for 1 h. The resulting pellet was discarded and the supernatant was filtered and loaded onto a 5-ml His-Trap chelating column (GE Healthcare, St. Gibes, UK). Proteins were eluted by applying a 10–500-mM imidazole gradient in buffer A. The proteins were dialyzed against buffer B (50-mM Hepes pH 7.9, 300-mM NaCl, 1-mM dithiothreitol and 10% [v/v] glycerol), aliquoted and stored at −80°C.

Full-length GtrS is a transmembrane protein and was present in the membrane fraction of the *E. coli* lysate. We generated membranes enriched in recombinant GtrS, for which cells were lysed using French Press treatment. After a first centrifugation at 5000 x g to remove whole cells, the supernatant was centrifuged at 20 000 x g for 30 min. The resulting pellet was resuspended in buffer B and used as an enriched GtrS fraction for analyses.

### Isothermal titration calorimetry

Microcalorimetric titrations were carried out at 25°C using a VP-microcalorimeter (Microcal, Amherst, Massachusetts, USA). GltS-LBD was dialyzed against 50-mM Hepes, pH 7.9, 300-mM NaCl, 1-mM dithiothreitol and 10% (v/v) glycerol and ligand solutions were made up in dialysis buffer. The titration involved 3.2-μM injections of 1–20-mM effector solutions into 20-μM GtrS-LBD. Control experiments involved the injection of effector solution into dialysis buffer. Raw titration data were concentration-normalized and corrected for dilution effects prior to analysis using the ‘One-binding site model’ of the MicroCal version of ORIGIN. The parameters Δ*H* (reaction enthalpy), *K*_A_ (binding constant, *K*_A_ = 1/*K*_D_) and *n* (reaction stoichiometry) were determined from the curve fit. The changes in free energy (Δ*G*) and entropy (Δ*S*) were calculated from the values of *K*_A_ and Δ*H* with the equation: Δ*G* = −*RT* ln *K*_A_ = Δ*H* − *T*Δ*S*, where *R* is the universal molar gas constant and *T* is the absolute temperature (23).

### Electrophoretic mobility shift assays

The P*_glk_*, P*_oprB_*, P*_edd/gap-1_* and P*_toxA_* promoter regions of *P. aeruginosa* PA01 were amplified by PCR using pMBL-T:P*_glk_*, pMBL-T:P*_oprB_*, pMBL-T:P*_edd/gap-1_* and pMBL-T:P*_toxA_* as templates respectively, and the set of primer pairs indicated in Supplementary Table S1. Amplified fragments were isolated from agarose gels and end-labeled with [γ-^32^P] deoxy-adenosinetriphosphate (ATP) using T4 polynucleotide kinase. A 10-μl sample containing 2 nM of labeled DNA (1.5 × 10^4^ cpm) was incubated with increasing concentrations of purified GltR for 1 h in 10 μl of binding buffer (50-mM Tris–HCl, pH 7.5, 10-mM NaCl, 0.5-M magnesium acetate, 0.1-mM EDTA; 1-mM DTT (Dithiothreitol), 5% [vol/vol] glycerol) containing 20 μg/ml of polyd(IC) and 200 μg/ml of bovine serum albumin. DNA–protein complexes were resolved by electrophoresis on 4% (wt/vol) non-denaturing polyacrylamide gels in 1 x TBE (Tris/Borate/EDTA: 89-mM Tris, 89-mM boric acid and 2-mM EDTA) using BioRad electrophoresis equipment as previously described (24).

### DNAse I footprinting

The DNA fragment containing P*_edd/gap-1_* and P*_oprB_* of *P. aeruginosa* PA01 was amplified as outlined above. DNA was labeled with [γ-^32^P] deoxy-ATP and 10-μl samples containing 2 nM of probe were mixed with different amounts of GltR (2, 4 and 10 μM) in binding buffer for the formation of the DNA–GltR complex. Samples were incubated at 30°C for 30 min, followed by the addition of DNAse I (0.4 U; Roche Biochemicals). After incubation for 2 min, the reaction was stopped by the addition of 2-μl 500-mM EDTA. DNA was extracted with phenol-chloroform, ethanol precipitated and dissolved in 10 μl of sequence loading buffer. After incubation at 90°C for 5 min, the DNA was loaded onto a 6.5% (wt/vol) DNA sequencing gel as described in (25). Appropriate sequencing reactions were loaded onto the gels along with the footprinting samples and used as a size ladder for identification of protected sites.

### Phosphorylation assays

#### *Autophosphorylation of GtrS*-Cter

Assays were carried out in 50-mM Hepes pH 7.9, 300-mM NaCl, 1-mM dithiothreitol, 2-mM MgCl_2_ and 10% [v/v] glycerol. The autophosphorylation was performed on ice with 30-μM purified GtrS-Cter in a final reaction volume of 100 μl. Reactions were started by adding radiolabeled ATP (0.2-mM ATP containing 10 μCi [γ-^32^P] ATP), and 10-μl samples were removed at different times. The reaction was stopped by the addition of 2 x sodium dodecylsulphate (SDS) loading buffer. Samples were analyzed on 12% (wt/vol) sodium dodecylsulphate-polyacrylamide gel electrophoresis (SDS-PAGE) gels.

#### Transphosphorylation from GtrS-Cter to GltR-MBP

GtrS-Cter (40 μM) was incubated with 0.2-mM ATP containing 10 μCi [γ-^32^P] ATP in the above buffer for 40 min for autophosphorylation. GltR-MBP at a final concentration of 8 μM was then added and samples were removed at regular intervals for analysis on 12% (wt/vol) SDS-PAGE gels.

### *In vitro* transcription assays

The P*_oprB_*, P*_toxA_* and P*_gap-1_* promoter regions of *P. aeruginosa* PAO1 were amplified by PCR using pMBL:P*_oprB_*, pMBL:P*_toxA_* and pMBL:P*_gap-1,_* respectively. *In vitro* transcription reactions (20 μl) were performed in 50-mM Tris–HCl (pH 7.5) supplemented with 10-mM NaCl, 0.5-M magnesium acetate, 0.1-mM EDTA and 1-mM DTT containing 50-nM σ^70^-holoenzyme, 20 units of RNasin (Promega) and 5-nM linear promoter DNA template that had been generated by PCR amplification as described above. The mixtures were incubated for 20 min at 30°C prior to the addition of Ribonucleoside Triphosphate Set (0.1-mM ATP, 0.1-mM CTP, 0.1-mM GTP, 0.05-mM UTP )and 3.6 μCi of [α-^32^P]UTP (10 μCi/μl) (1 Ci = 37 GBq). The cytosolic fragment of GtrS (GtrS-C-ter) or full-length GtrS was added to these samples along with GltR, PtxR or HexR depending on the experiment. After 30-min incubation, the reactions were chilled at 4°C, and 4 μl of formamide sequencing dye was added. Samples were separated on a 6.5% (wt/vol) PAGE gels. When indicated, ATP or 2-KG was added to the reaction mixture at the indicated concentrations.

## RESULTS

### 2-KG binds with high affinity to the recombinant ligand-binding domain of GtrS

Analysis of the 473 amino acid protein GtrS by Pfam resulted in the identification of HAMP (PF00672), HisKA (PF00512) and HTPAse_c (Pfam 02518) domains which are characteristic features of sensor kinases. Using the DAS algorithm (26), two transmembrane regions were detected (amino acids 8–22 and 200–217, Supplementary Figure 1). The 177 amino acid sequence fragment flanked by these putative transmembrane domains was assumed to form the periplasmic ligand-binding domain. However, this fragment remains unannotated in all relevant databases. In addition, homology and remote modeling attempts failed and no significant sequence similarity was observed for this fragment with proteins present in the SwissProt database of characterized proteins.

**Figure 2.**
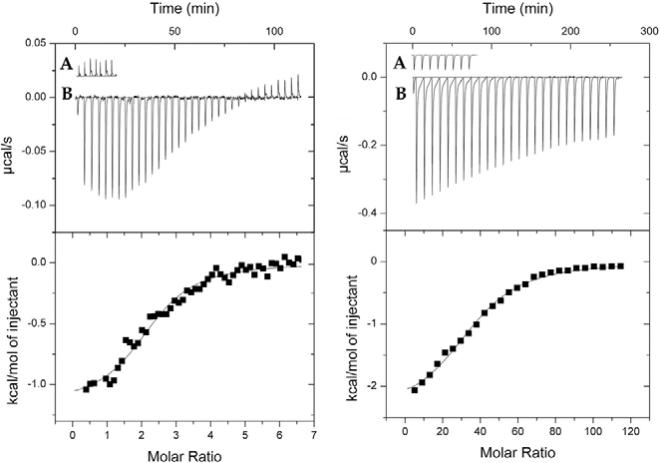
Microcalorimetric binding studies of effectors to the recombinant ligand-binding region of GrtS (GrtS-LBD). Left panel: titration raw data for the injection of 3.2-μl aliquots of 1-mM 2-KG into dialysis buffer (**A**) and 20-μM GrtS-LBD (**B**). Right panel: titration raw data for the injection of 3.2-μl aliquots of 1-mM 6PG into dialysis buffer (**A**) and 20 μM of GrtS-LBD (**B**). The dilution-corrected and concentration-normalized peak areas for the protein titration are shown in the lower panels. Data were fitted with the ‘One-binding site model’ of the MicroCal version of ORIGIN.

To explore which ligands, if any, bind to this putative binding domain, the corresponding DNA fragment was cloned into an expression vector and the recombinant ligand-binding domain (GtrS-LBD) was expressed in *E. coli* and purified from the soluble cell lysate by affinity chromatography. In previous studies of glucose regulators in *Pseudomonas*, isothermal titration calorimetry assays were essential to identify the specific effector molecules recognized by these regulators. In fact, PtxS and HexR were found to bind 2-KG and KDPG, respectively, which are intermediates of glucose metabolism. We then submitted GtrS-LBD to microcalorimetric titrations with all possible intermediates of glucose metabolism (Supplementary Figure S2). As shown in Figure 2, binding was observed for two ligands, namely, 2-KG and 6PG. Data analysis for the binding of 2-KG revealed a *K*_D_ of 4.95 ± 0.8 μM in a reaction driven by favorable enthalpy (Δ*H* = −1.16 ± 0.1 kcal/mol) and entropy (*T*Δ*S* = 5.95 ± 0.1 kcal/mol) changes. The binding of 6PG was characterized by a *K*_D_ of 98 ± 33 μM, which implies that the affinity for this compound is around 20-fold weaker than that for 2-KG.

### The cytosolic fragment of GtrS shows autokinase activity

**Figure 3.**
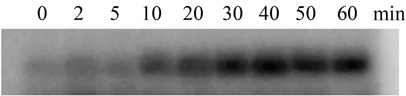
Autophosphorylation activity of the C-terminal segment of GtrS. The recombinant protein GtrS-Cter comprising amino acids 269–465 of GtrS was incubated with 0.2-mM ATP containing 10 μCi of [γ-^32^P] ATP. Aliquots were removed at the times indicated for analysis by SDS-PAGE.

To verify whether GtrS has autokinase activity, the DNA sequence encoding amino acids 269–465, corresponding to the C-terminal and cytosolic part of the protein, was cloned into the pET28a(+) expression vector. The recombinant protein termed GtrS-Cter was expressed in *E. coli* and purified using affinity chromatography. To detect protein autophosphorylation, GtrS-Cter was incubated with 0.2-mM ATP containing 10 μCi of [γ-^32^P] ATP. At different time points, samples were removed for analysis by SDS-PAGE. As shown in Figure 3, an increase in the amount of phosphorylated GtrS-Cter was observed with time, which provides evidence for autophosphorylation activity.

### GltR binds to promoters of genes for glucose metabolism and transport, and expression of exotoxin A

**Figure 4.**
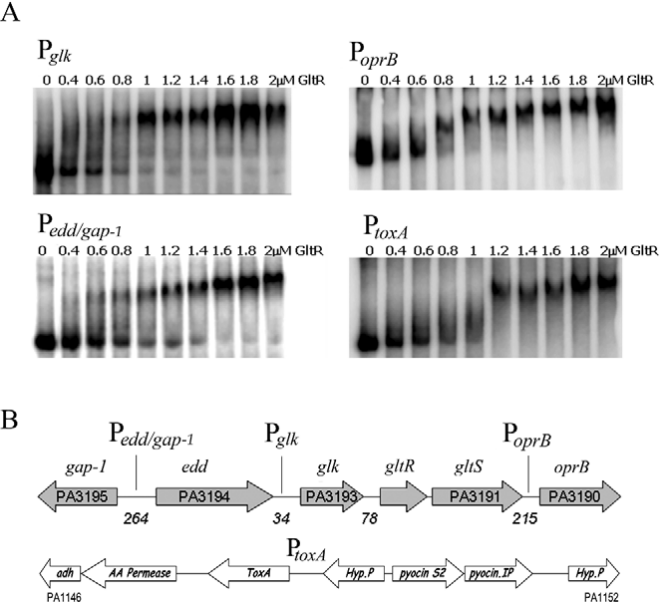
Interaction of GltR with promoter regions. (**A**) EMSAs for the binding of GltR to different regions. DNA fragments from promoters P*_glk_*, P*_edd/gap-1_*, P*_oprB_* and P*_toxA_* were used. Protein binding was tested in the range 0–2 μM. (**B**) Schematic representation of the promoters at which GltR was found to bind. The gene names as well as the size (bp) of the intergenic regions are indicated.

According to Pfam, the 242 amino acid protein GltR is composed of an N-terminal receiver domain (PF00072) and a C-terminal winged helix-turn-helix DNA-binding domain (PF00486). GltR belongs to the OmpR/PhoB family of response regulators, which is the most abundant response regulator family present in all bacterial phyla (27). To identify promoters that are recognized by GltR, the purified recombinant protein was submitted to electrophoretic mobility shift assays (EMSAs) using DNA probes of eight different promoters. As shown in Figure 4, GltR was found to bind to four promoters that are involved in glucose metabolism. Three promoters, namely P*_glk_*, P*_edd_*_/_*_gap-1_* and P*_oprB_*, control the expression of *glk*, phosphogluconate dehydratase (*edd*), *gap-1* and a fourth one was found upstream of the ORF encoding the OprB protein that is predicted to be part of an ABC-type sugar transporter (Figure 1). Since we have previously shown that PtxR, but not HexR, binds to the complex P*_toxA_* promoter, we also tested binding of GltR to the *toxA* promoter. We found that GltR binds to P*_toxA_* as it retarded the DNA migration in EMSA (Figure 4). GltR bound to all of these promoters with an affinity of ∼1 μM. The promoters that were analyzed but for which no binding was observed included promoters P*_kgu_*, P*_gad_*, P*_ompR_* and P*_mnzV_* (Supplementary Figure S3A).

### Identification of the DNA-binding sequence for GltR

**Figure 5.**
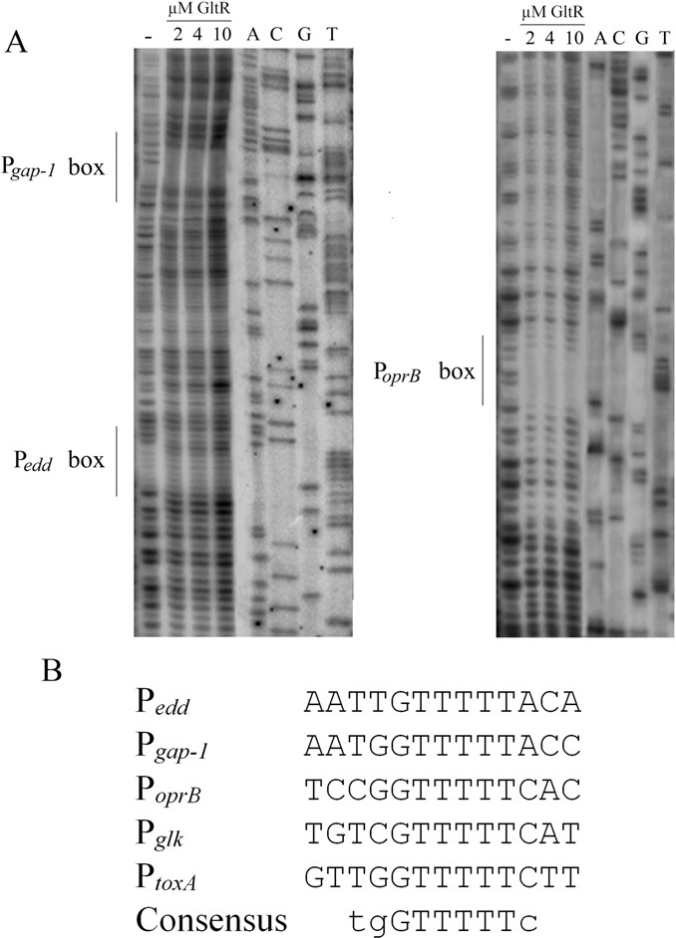
Determination of the GltR operator site. (**A**) DNAse I footprint experiments with a DNA sequence of promoter P*_edd-gap-1_* (left) and P*_oprB_* (right). The DNA fragment protected by GltR is highlighted. (**B**) Sequence alignment of the boxes P*_edd_*, P*_gap-1_* and P*_oprB_* as determined above with sequence fragments present in promoters P*_kgl_* and P*_toxA_* that were shown by EMSA to bind GltR (see Figure 4).

To identify the GltR operator sites, DNAse I footprinting assays were carried out using the P*_edd_*_/_*_gap-1_* and P*_oprB_* promoters (Figure 5). Two GltR operator sites were identified in the DNA fragment comprising P*_edd_* and P*_gap-1_*. These sites are proposed to correspond to two overlapping promoters that control the divergently transcribed *edd* and *gap-1* genes (Figure 4B). A single operator site was found to be present in the promoter P*_oprB_* (Figure 5A). To verify this result, an EMSA with the P*_oprB_* fragment was conducted, in which the identified operator site was replaced by a random sequence. As shown in Supplementary Figure 3B, mutation of this site resulted in failure of GltR to bind. The alignment of the three operator sites identified (Figure 5B) within the promoter sequences of P*_glk_* and P*_toxA_*, the two other promoters to which GltR binds, resulted in the 5’-tgGTTTTTc-3’ consensus sequence for GltR binding (Figure 5B).

### GtrS and GltR form a two-component system and 2-KG increases GtrS autophosphorylation

**Figure 6.**
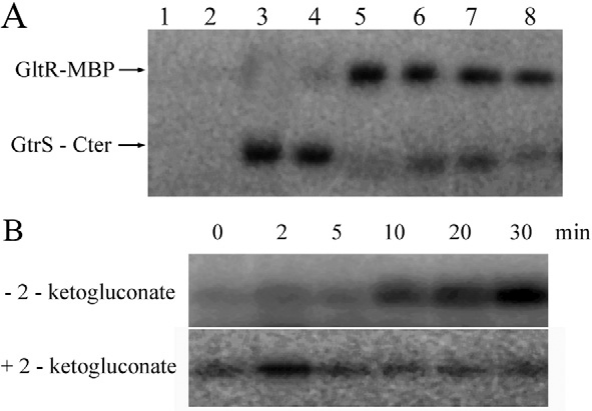
GtrS and GltR form a TCS and 2-ketoglucoate modulates GtrS autokinase kinetics. (**A**) Transphosphorylation between the GltR-MBP fusion and the cytosolic part of GtrS (GtrS-Cter) comprising its autokinase domain. Lane 1: incubation of GltR-MBP with [γ-^32^P] ATP for 40 min; Lane 2 and 3: incubation of GtrS-Cter with [γ-^32^P] ATP for 0 and 40 min, respectively; Lanes 4–9: GltR-MBP was added to phosphorylated GtrS-Cter and samples removed for SDS-PAGE after 0 min (lane 4), 2 min (lane 5), 5 min (lane 6), 15 min (lane 7) and 30 min (lane 8). (**B)** Effect of 2-KG on autophosphorylation activity of full-length GtrS. A suspension of *E. coli* membranes containing full-length GtrS was incubated with 0.2-mM ATP containing 10 μCi [γ-^32^P] ATP in the absence and presence of 2-KG. Aliquots were removed for analysis by SDS-PAGE at the times indicated.

To determine whether the two regulator proteins GtrS and GltR form a TCS, transphosphorylation assays were performed. These assays require that the two proteins differ in their electrophoretic mobility. Since GltR and GrtS-Cter are of similar size, they were found to co-migrate on SDS-PAGE gels. To be able to differentiate them, we fused GltR with the MBP; this led to an increase in the molecular weight of the recombinant GltR by ∼43 kDa. Initial experiments revealed that ATP does not phosphorylate GltR-MBP (Figure 6A, lane 1), whereas GtrS-Cter was efficiently phosphorylated by this phosphodonor, and labeled protein was seen as soon as 2 min after initiation of the incubation (lanes 3 and 4, Figure 6A). The addition of GltR-MBP to phosphorylated GtrS-Cter caused an efficient transphosphorylation of GltR-MBP, which appeared to be complete following a contact time of 2 min. This, combined with the concomitant disappearance of phosphorylated GtrS-Cter, can be considered as proof that GtrS and GltR form a TCS.

Subsequent experiments were aimed at determining the effect of 2-KG on the kinetics of GtrS autophosphorylation. Full-length GtrS was overexpressed in *E. coli* and the membranes enriched with this protein were purified. The incubation of these membranes with [γ-^32^P] ATP resulted in a gradual increase in the phosphorylation state of GtrS. However, in the presence of 2-KG, a rapid increase in the phosphorylation state of this protein was observed (Figure 6B). The subsequent reduction in the GtrS phosphorylation state is likely due to GtrS phosphatase activity. It can be concluded that 2-KG increases the autophosphorylation kinetics of GtrS.

### 6-Phosphogluconate and 2-KG relieve the repression exerted by the GtrS/GltR TCS

In an effort to further investigate the observation that 2-KG and 6PG bind to the recombinant ligand-binding region of GtrS, we conducted gene expression studies. To this end, the *gap-1, edd, oprB*, *glk* and *toxA* promoters were fused to the ‘*lacZ* reporter gene and the resulting plasmids were introduced into *P. aeruginosa*. As detailed in Table 2, the basal expression from these promoters in the absence of added ligands were in the range of 100–450 Miller Units. The addition of 2-KG resulted in an increase in transcriptional activity from these promoters by a factor of ∼3. Similar results were observed when 6PG was used as an effector, although the magnitude of the transcriptional increase was somewhat inferior to that observed for 6-ketogluconate. This difference may be related to the higher affinity of GtrS for 2-KG.

**Table 2.** Expression from promoters P*_edd/gap-1_*, P*_oprB_*, P*_glk_* and P*_toxA_* in the wild-type *P. aeruginosa* and its isogenic *gltR* mutant measured as β-galactosidase activity

Host	Promoter	No effector	+ 2-KG	+ 6-PG
Wild-type	P*_gap-1_::lacZ*	120 ± 20	355 ± 30	255 ± 10
Wild-type	P*_edd_::lacZ*	470 ± 40	960 ± 15	860 ± 20
Wild-type	P*_oprB_: :lacZ*	370 ± 20	930 ± 10	840 ± 10
Wild-type	P*_glk_::lacZ*	100 ± 10	530 ± 11	435 ± 20
Wild-type	P*_toxA_::lacZ*	240 ± 20	1370 ± 70	1160 ± 30
Δ*gltR*	P*_gap-1_::lacZ*	310 ± 20	480 ± 10	375 ± 20
Δ*gltR*	P*_edd_::lacZ*	1265 ± 10	1270 ± 10	1155 ± 10
Δ*gltR*	P*_oprB_: :lacZ*	780 ± 20	1050 ± 15	980 ± 15
Δ*gltR*	P*_glk_::lacZ*	350 ± 50	480 ± 20	375 ± 10
Δ*gltR*	P*_toxA_::lacZ*	1450 ± 20	1210 ± 30	1015 ± 20

Experiments were conducted in the presence and absence of effectors of GtrS, namely, 2-KG and 6-PG. Activities are expressed in Miller Units.

To evaluate the contribution of the GtrS/GltR TCS in this regulatory process, an isogenic *gltR* mutant was constructed and the experiments described above were repeated in this mutant background. Interestingly, the average basal transcriptional activity of the mutant in the absence of effector almost coincides with the activity observed for the wild-type strain in the presence of 2-KG (Table 2). The addition of either 2-KG or 6PG to the mutant strain did not cause any significant changes in the transcriptional activity. These data demonstrate that the GtrS/GltR TCS represses transcription from these five promoters and this repression is entirely relieved by the addition of 2-KG or 6PG.

### Transcription repression exerted by GltR/GtrS system

**Figure 7.**
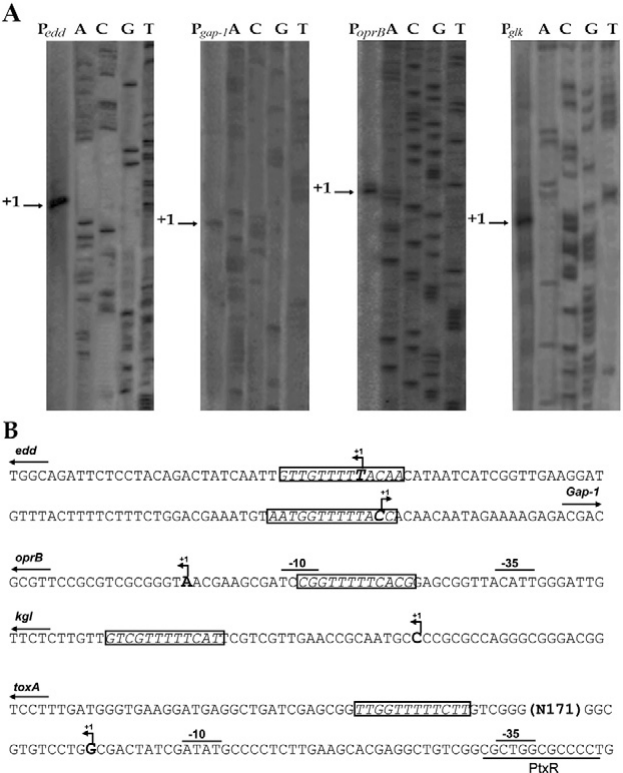
Analysis of the P*_edd-gap-1_*, P *_oprB_*, P*_glk_* and P*_toxA_* promoters. (**A**) Determination of the transcription start point using primer extension analysis. (**B**) Sequences of the promoters regulated by the GltR transcriptional regulator. The transcriptional start sites are indicated by arrows and bold letters. Boxes highlight the GltR-binding sites and the -10 and -35 binding sites for the RNA polymerase are indicated.

Our gene expression data showed that the GtrS/GltR TCS modulates expression from the promoters P*_glk_*, P*_edd/gap-1_*, P*_oprB_* and P*_toxA_*. We studied the mechanism by which GltR mediates this effect determining the transcription start point(s) for each of the promoters (Figure 7A). The inspection of the promoter sequence showed that the GltR operator is at position −4 to +9, −11 to +2, −12 to −23, +21 to +33 and +188 to +200 relative to the transcriptional initiation site of the *edd, gap-1, oprB, glk* and *toxA* promoters, respectively (Figure 7B). These results suggest that binding of GltR either inhibits RNA polymerase binding or the advancement of the active transcriptional complex. It should be noted that at P*_toxA_*, a second GltR-binding site is further downstream of the *tsp* (+188 to +200) and its role might be to guarantee blockage of the advance of the RNA polymerase complex.

### Phosphorylation of GltR induces its dissociation from DNA leading to transcriptional activation

**Figure 8.**
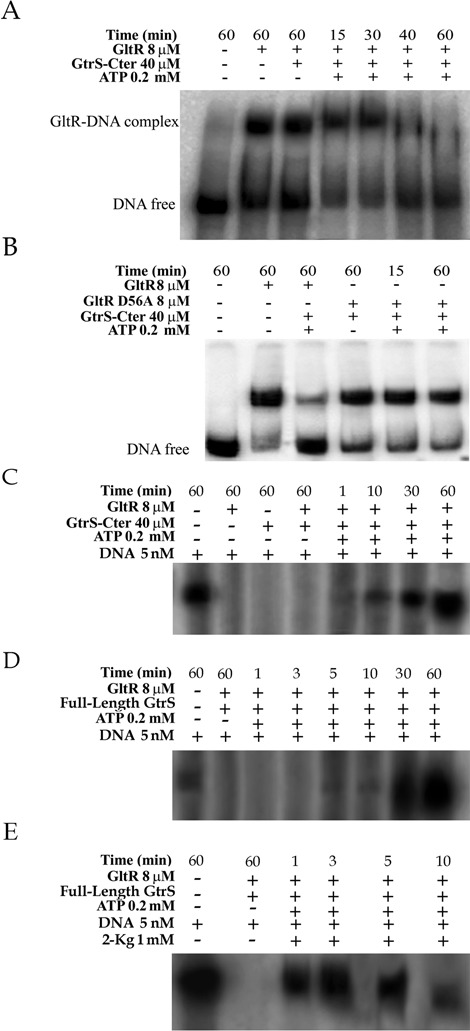
Phosphorylation of GltR induces its dissociation from DNA causing transcriptional activation. (**A** and **B**) EMSAs of a DNA fragment harboring P*_oprB_* in the presence of wild-type GltR (A) or the D56A mutant of GltR (B). Where indicated, samples were incubated with 0.2-mM ATP for the times indicated prior to analysis by SDS-PAGE. (**C**–**E**) *In vitro* transcription assays were carried out as described in Materials and Methods. (C) Transcription from P*_oprB_* mediated by the cytosolic fragment of GtrS (GtrS-C-ter) and GltR in the absence and in the presence of ATP. Experimental conditions and protein concentrations were similar to those of part (A). Where indicated, samples were incubated with 0.2-mM ATP for different intervals prior to electrophoretic analysis. (**D** and **E**) Transcription with membranes containing full-length GtrS (2 μg/μl of total protein), GltR and ATP in the absence (D) and in the presence of 2-KG (E). Experimental conditions were identical to those of part (B).

We have shown so far that GtrS effector molecules increase GltR autophosphorylation and that this increase is translated into transcriptional activation. To determine the molecular mechanism of this process in more detail, we conducted experiments to evaluate the effect of GltR phosphorylation on its DNA-binding capacity. To this end, EMSAs were conducted and the results are shown in Figure 8. As shown above, non-phosphorylated GltR bound to DNA and the addition of GtrS-Cter (in the absence of ATP) did not alter this interaction (Figure 8A, lanes 2 and 3). However, when the DNA/GtrS-Cter/GltR complex is incubated with ATP, a gradual release of GltR is observed (Figure 8A). As shown earlier, ATP causes autophosphorylation of GtrS and concomitantly transphosphorylation of GltR, and the GltR-P form is released from DNA. Subsequently, this experiment was repeated with the GltR mutant, in which the phosphorylgroup-accepting aspartate 56 was replaced by alanine and which has, therefore, lost its capacity to get phosphorylated. As shown in Figure 8B, the incubation of this mutant protein with GtrS-Cter and ATP did not cause protein dissociation from DNA. Taken together, it can be concluded that phosphorylation at D56 of GltR causes the release of DNA-bound protein. We have also investigated whether GltR can be phosphorylated by the low molecular weight phosphodonors acetylphosphate and carbamoylphosphate and have conducted microcalorimetric experiments similar to those described for the TodT response regulator (28). However, no reaction heats could be monitored indicating that GltR is among the response regulators that are not phosphorylated by low molecular weight donors.

To verify whether this ATP-mediated dissociation of GltR results in transcriptional activation, *in vitro* transcription assays were conducted. Initial experiments (Figure 8C) were carried out using a similar experimental set up as the EMSA shown above. No transcription is observed in the presence of GltR or the GtrS-Cter/GltR in the absence of ATP. However, transcription is initiated by the addition of ATP and continues to increase steadily. These data show that GltR phosphorylation leads to its dissociation, causing repression relieve of and enabling transcription.

### 2-KG dramatically enhanced transcription

To assess the effect of 2-KG on transcriptional activity, experiments were performed with membranes enriched in full-length GtrS. In the absence of 2-KG, only low levels of transcription were seen after 5 min; the transcription increased to its maximum following 60-min incubation (Figure 8D). When this experiment was repeated in the presence of 2-KG (Figure 8E), a strong band was seen after 1 min and the transcript level after 3 min was comparable to that seen after 60 min in the absence of 2-KG. These results support the idea that 2-KG enhances GtrS autophosphorylation and in turn stimulates GltR phosphorylation leading to its dissociation from the DNA enabling transcription.

### Complexity in the transcriptional regulation of P*_toxA_* and P*_gap-1_*

**Figure 9.**
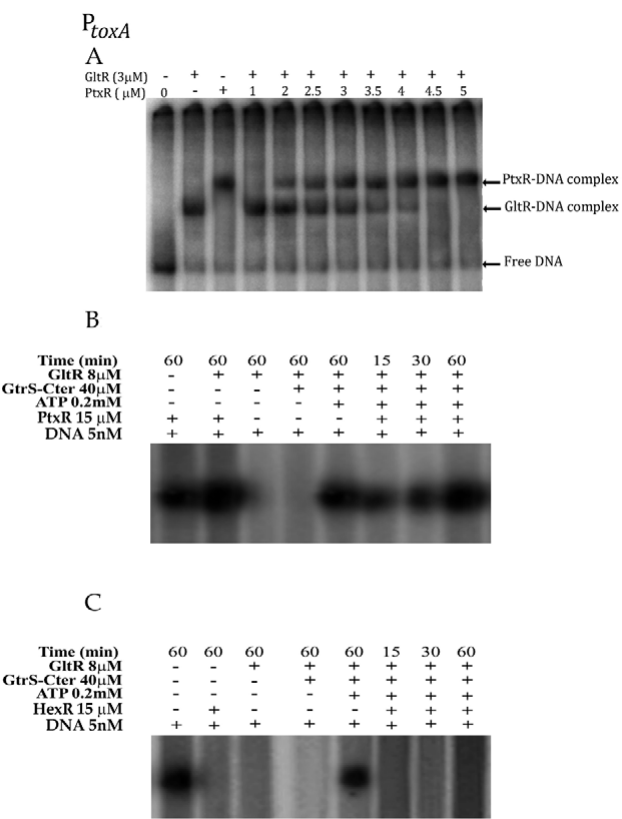
Complexity of transcriptional regulation of promoters P*_toxA_* and P*_gap-1_* by the concerted action of GtrS/GltR and PtxR or HexR, respectively. (**A**) EMSAs of a DNA fragment harboring P*_toxA_* in the presence of either GltR, PtxR or both regulators. (**B**) *In vitro* transcription assays from P*_toxA_* of the GtrS-Cter/GltR system in the presence and absence of PtxR. (**C**) *In vitro* transcription assay from P*_gap-1_* of the GtrS-Cter/GltR system in the presence and in the absence of HexR.

The regulation of *toxA* gene expression is extremely complex, since a number of different regulator proteins, namely, RegA, PtxR, Vfr, Fur, PvdS, PtxR and PtxS have been shown to be involved (8,29,30). In this work, we show that P*_toxA_* expression is also regulated by the GltR/GltS TCS (Supplementary Figure S4). To explore possible mutual interactions between this TCS and the PtxR regulator, we conducted EMSAs (Figure 9A) with a DNA fragment that harbors the PtxR operator (covering the -35 region) as well as the GltR operator, which is ∼215 bp downstream of the PtxR-binding site (Figure 7 and Supplementary Figure S4B). EMSAs with a constant amount of GltR, but increasing amounts of PtxR (Figure 9A) clearly showed that the band corresponding to bound GltR diminished proportionally to the increase in the band corresponding to bound PtxR. Interestingly, no band corresponding to the DNA fragment bound to both proteins could be detected, which shows that binding of either protein is exclusive and is suggestive of competition. This was unexpected since the operator sites are spaced out by some 200 bp. *In vitro* transcription assays (Figure 9B) show that the GtrS-Cter/GltR system is a repressor, whereas PtxR is an activator.

As shown in Supplementary Figure S4, the expression of the P*_gap-1_* promoter is controlled by HexR and the GtrS/GltR system. As illustrated in Figure 9C, both systems can on their own repress transcription from P*_gap-1_*. However, transcriptional activation by the addition of ATP causing GltR phosphorylation is only observed in the absence of HexR, whereas no transcription is observed in the presence of HexR, suggesting a dominating role of this repressor in the control of the expression from P*_gap_*_-1_.

## DISCUSSION

Bacteria of the genus *Pseudomonas* are microorganisms with a robust metabolism for aromatic compounds, amino acids and organic acids; however, the number of sugars metabolized by these microbes is rather limited and mainly restricted to glucose, gluconate and fructose. This metabolic pattern has been associated with their lifestyle as environmental microbes exposed to a limited number of sugars. Conservation of the glucose catabolic genes in *Pseudomonas* could be related to their association with plant roots, where glucose is an abundant food source (31,32).

The current knowledge on the glucose metabolism in *Pseudomonas* is summarized in Figure 1. The relevance of this complexity is deduced from several facts: (i) Multiple glucose degradation pathways exist and, in spite of their redundancy, they work simultaneously to produce 6PG, which is metabolized via the Entner–Doudoroff pathway. (ii) Genes are organized in operons made of ORFs that encode enzymes for different catabolic segments; this guarantees that all glucose metabolism pathways are induced simultaneously. The structure of glucose degradation operons with genes of different pathways in different operons can be regarded as relevant to guarantee balanced utilization of sugars. (iii) Catabolic segments are under the control of several regulators that respond differentially to distinct pathway intermediates. (iv) There is a hierarchy in the control of the expression of glucose metabolism in *Pseudomonas*.

The results presented in this study support that several promoters of the glucose degradation operons, namely, *gap-1, edd, oprB and glk* are under the control of a newly identified TCS, and this system also controls the expression of the virulence factor *toxA*.

These results raise a number of questions: Why are a multitude of regulatory systems required to control glucose metabolism? Why is a hierarchy in place to control these pathways when in the environment microbes are exposed to multiple C-source? The reason may lie in the fact that the genes that encode the enzymes are metabolically convergent. The PtxS repressor dominates over positive action exerted by PtxR and PtxS is also dominant over the GtrS/GltR system. The metabolic advantage is that while PtxS just recognizes 2-KG, GtrS recognizes both 2-KG and 6PG. Thus, the dual control guarantees the response to more than one compound.

Del Castillo *et al.* (16) showed that the homologous GltR-2 in *P. putida* KT2440 is a transcriptional activator involved in the induction of the glucose transport system and is not a repressor of the glucose metabolism, which contrasts with the GltR function in *P. aeruginosa*. These differences may be explained by data that suggest that the GtrS/GltR systems in *P. aeruginosa* and *P. putida* operate on different regulons. We show here that the system modulates expression from promoters *toxA, gap-1, edd, oprB* and *glk*. Apart from the fact that *P. putida* KT2440 has no *toxA* equivalent, the genes homologous to those regulated by GtrS/GltR in *P. aeruginosa* were not identified in the transcriptomics study of del Castillo *et al.* (16) comparing transcription in a *gltR* mutant with that of the wild-type strain.

Another important issue in this regulation cascade is the affinity of regulators for effectors. Both, PtxS and GtrS, recognize 2-KG with similar affinities, while GtrS recognizes metabolites downstream of 2-KG with lower affinity. This could have evolved in order to guarantee metabolism in cases where large amounts of 2-KG is found in the environment or when *P. putida* is a member of a microbial community and the strain is neighbored by microbes producing 6PG.

Prototypical TCSs are made of a histidine protein kinase (HK), containing a conserved kinase core, and a response regulator protein (RR), containing a conserved regulatory domain (33). As the number of characterized TCSs grows, the inventory of variations on the basic scheme also grows (34,35). In this study, we have shown that the GrtS/GltR regulators form a previously unidentified TCS. GrtS is an orthodox HK that has two transmembrane regions, which separate the protein into a periplasmic N-terminal sensing domain and a cytoplasmic C-terminal catalytic region that is designated as the kinase core. GrtS recognizes 2-KG and 6PG in the periplasm, where these chemicals are synthesized so that the physical structure of this kinase allows it to recognize a compound in the periplasm and transmit the signal to the cytoplasmic GltR protein. Binding of the effector to the HK (GrtS) stimulates autophosphorylation, which is subsequently transferred to the GltR response regulator protein. GltR appears to be the terminal component of the pathway, functioning as a phosphorylation-activated switch to affect the adaptive response.

The regulatory domains of RRs are thought to exist in equilibrium between two conformational states, inactive and active (36). In a positive transcriptional RR, phosphorylation of the regulatory domain shifts the equilibrium toward the active form. In this form, the RR can facilitate specific protein–DNA interactions or interactions with RNA polymerase or other regulators leading to the activation of transcription (37,38,39,40,41,42,43,44,45,46,47,48). GltR belongs to a small group of previously reported RRs, in which phosphorylation corresponds to the off state, a case described for the yeast osmoregulator (SSK1), whose phosphorylated form is the ‘off’ state (49). Thus, in the system we present here, phosphorylation of GltR leads to removal of the repressive effect.

In summary, this study presents an example of how a group of complex convergent catabolic pathways are controlled by a set of repressors and activators that respond to different chemicals in the pathways in order to guarantee the appropriate expression for balance growth.

## SUPPLEMENTARY DATA

Supplementary Data are available at NAR Online.

SUPPLEMENTARY DATA
