# Supplementary material for: GtrS and GltR form a two-component system: the central role of 2-ketogluconate in the expression of exotoxin A and glucose catabolic enzymes in Pseudomonas aeruginosa
Source: Nucleic Acids Res. 2014 Jun 11;42(12):7654–65. doi: 10.1093/nar/gku496 (PMC4081096; doi:10.1093/nar/gku496)
Supplement: SUPPLEMENTARY DATA [file supp_42_12_7654__index.html]

GtrS and GltR form a two-component system: the central role of 2-ketogluconate in the expression of exotoxin A and glucose catabolic enzymes in Pseudomonas aeruginosa — SUPPLEMENTARY DATA 

# GtrS and GltR form a two-component system: the central role of 2-ketogluconate in the expression of exotoxin A and glucose catabolic enzymes in *Pseudomonas aeruginosa*

## SUPPLEMENTARY DATA

**Files in this Data Supplement:**

- Supplementary Data
