# Supplementary material for: GtrS and GltR form a two-component system: the central role of 2-ketogluconate in the expression of exotoxin A and glucose catabolic enzymes in Pseudomonas aeruginosa
Source: Nucleic Acids Res. 2014 Jun 11;42(12):7654–65. doi: 10.1093/nar/gku496 (PMC4081096; doi:10.1093/nar/gku496)
Supplement: SUPPLEMENTARY DATA [file supp_gku496_nar-00527-x-2014-File011.docx]

**Supplementary data to**

#### GtrS and GltR form a two-component system: The central role of 2-ketogluconate in the expression of exotoxin A and glucose catabolic enzymes in

#### *Pseudomonas aeruginosa*

Abdelali Daddaoua, Carlos Molina-Santiago, Jesús de la Torre, Tino Krell and Juan-Luis Ramos


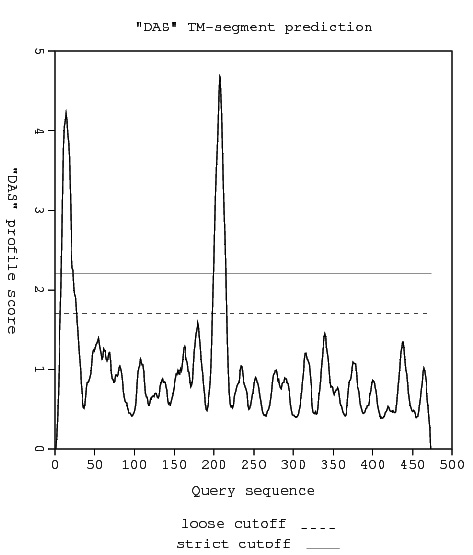


**Supp. Figure 1: Graphical output from the prediction of transmembrane helices in GtrS using the DAS algorithm (**Cserzo *et al*. (1997) Protein Engineering 10:673-676)**.** Peaks over a height of 2.2. are considered as reliable predictions.

**
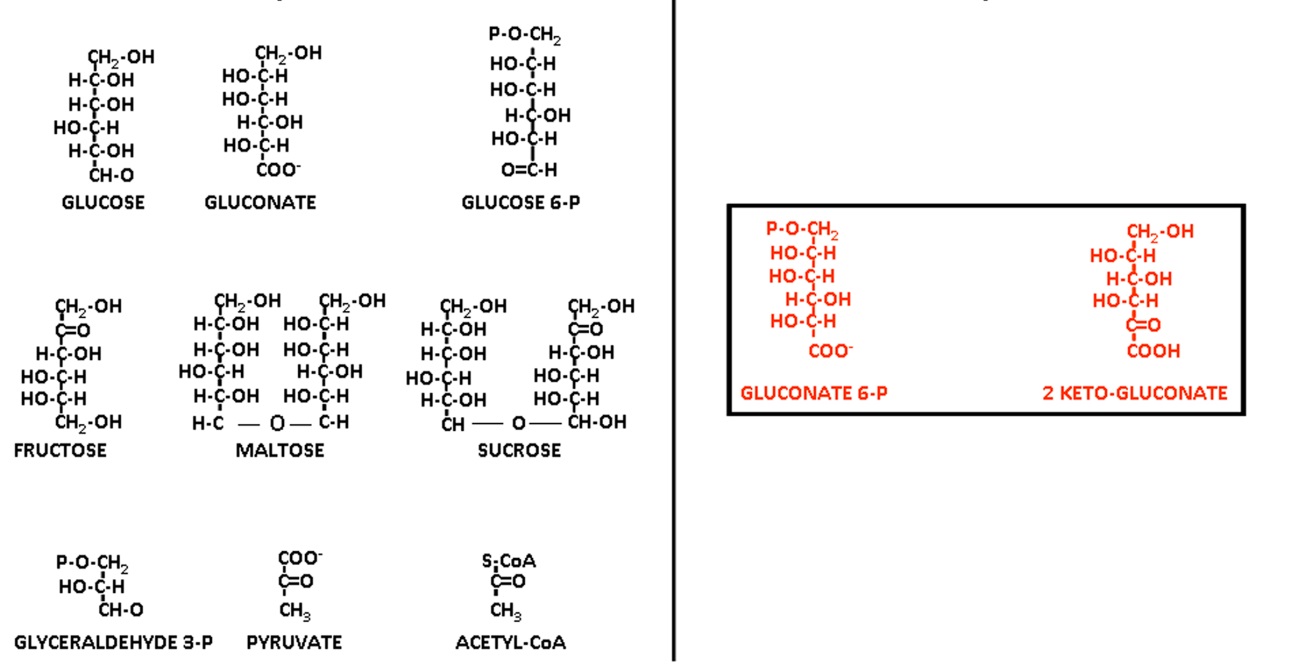
**

**Supp. Figure 2: Compounds used for microcalorimetric binding studies to the recombinant ligand binding domain of GtrS (GtrS-LBD).** Compounds shown in red showed binding, whereas compounds in black failed to bind.


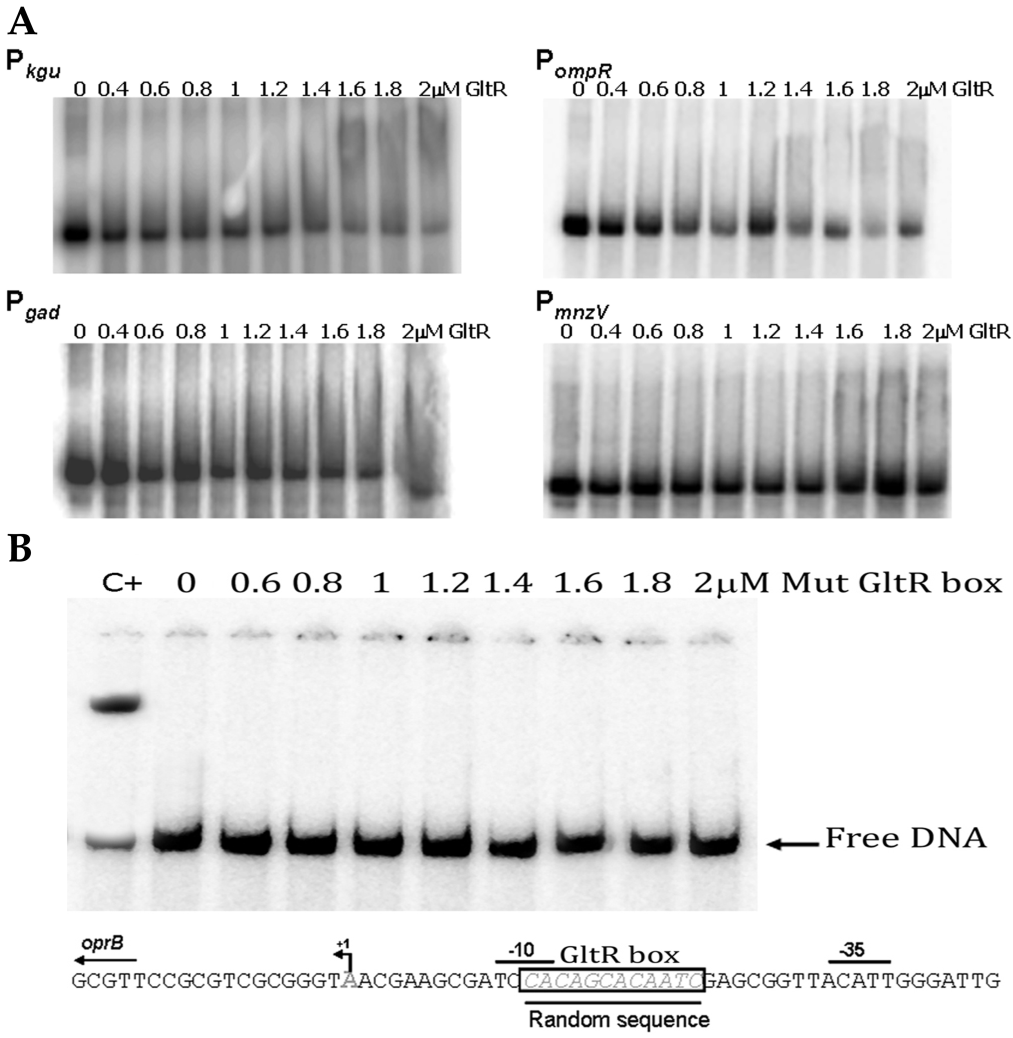


**Supp. Figure 3: GltR binding studies to different promoters. A) Electrophorectic mobility shift assays of different promoters with GltR.** In all cases an absence of binding was noted. **B):** EMSA of GltR and an *oprB*-variant operator region in which the boxed fragment was replaced by a random sequence. The DNA binding assays were carried out using 2 nM of the *oprB*-variant operator region amplified by PCR and end labelled with [γP^32^] and then incubated with increasing concentrations of GltR protein (from left to right: 0, 0.6, 0.8, 1, 1.2, 1.4, 1.6, 1.8 and 2 μM). The control positive (C+) indicate the binding of GltR protein to the native sequence of *oprB* promoter.

**
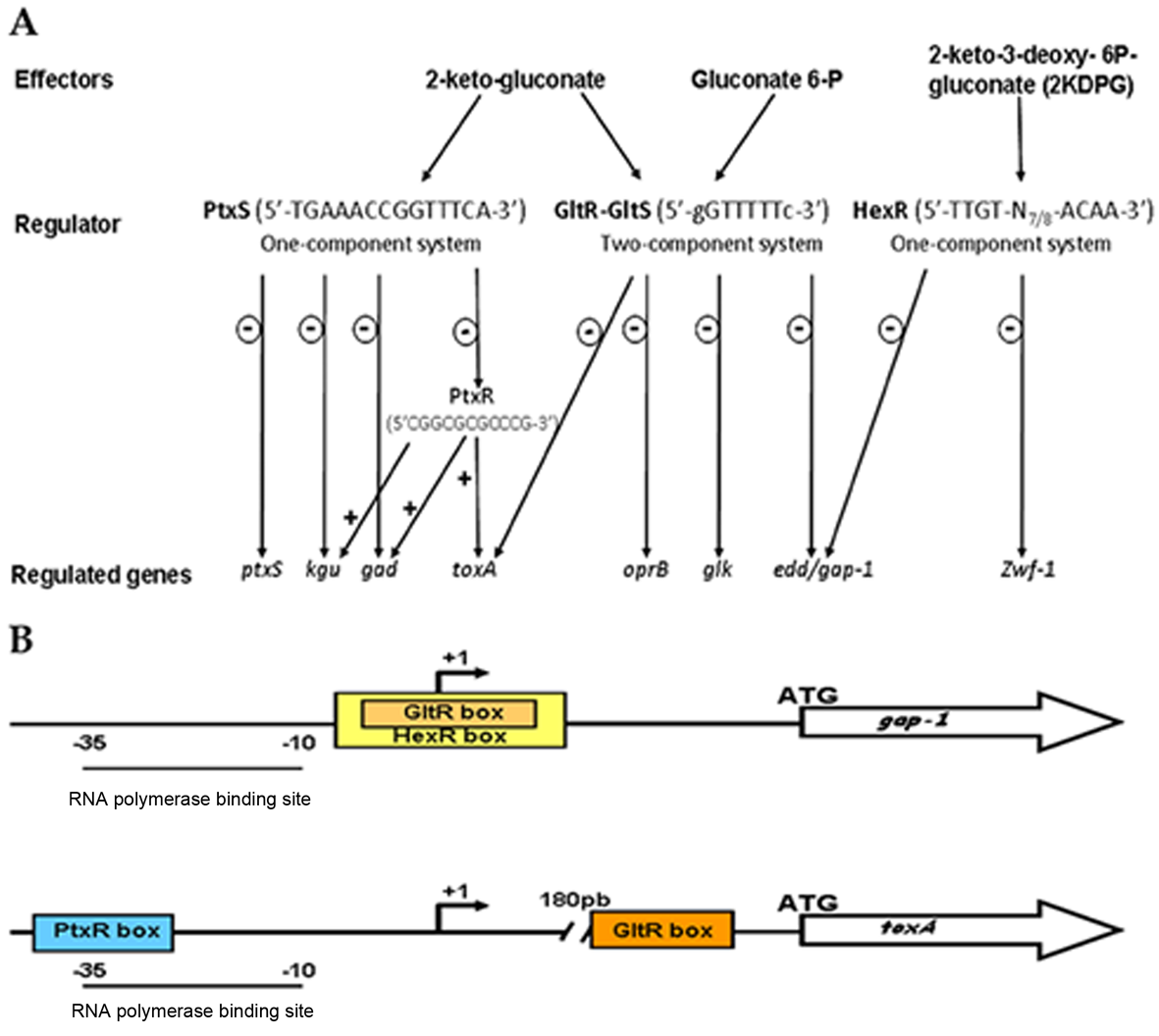
**

**Supp. Fig. 4: Complexity in the regulation of *toxA* and glucose metabolism genes in *P. aeruginosa* PAO1. A)** Summary of effectors and regulators that participate in the regulation of individual promoters. The consensus DNA sequence for the different regulators are shown in brackets. -, repression of transcription; + activation of transcription. **B)** Respective positions of GltR and HexR operators in the *gap-1* promoter and PtxR and GltR operators in the *toxA* promoter.

**Suppl. Table 1. Primers used in this study**.

| **Primer** | **Sequence** | **Purpose** |
| --- | --- | --- |
| ΔGltR.f | 5’-AGCCTGGCGATCCTCGACGT-3’ | Δ *gltR*::Gm generation |
| ΔGltR.r | 5’-GGTCGAGGATCTGCTGCGGAT-3’ | Δ*gltR*::Gm generation |
| GltR.f | 5’-CATATGATGACCAGGAAATCCG-3’ | Generation of recombinant GltR |
| GltR.r | 5’-GGATCCCCGTTCGCTCAT-3’ | Generation of recombinant GltR |
| GltR D56A f | 5’‑GCGAGCCTGGCGATCCTCGCGGTGATGCTGCCGGACGAGGAC –3´ | Generation of recombinant GltR D56A |
| GltR D56A r | 5´‑CTCGTCCGGCAGCATCACCGCGAGGATCGCCAGGCTCGCCTC-3´ | Generation of recombinant GltR D56A |
| GtrS.f | 5’-CATATGCGCATGCTGCTCCTGA-3’ | Generation of recombinant GtrS |
| GtrS.r | 5’-GGATCCTCACTCCAGCCCCAGG-3’ | Generation of recombinant GtrS |
| GtrS-Cter.f | 5’-CATATGCATTGGCAGAGCCGTCC-3’ | Generation of recombinant GtrS-Cter |
| GtrS-Cter.r | 5’-GGATCCTCACTCCAGCCCCAGG-3’ | Generation of recombinant GtrS-Cter |
| GtrS-Lbd.f | 5’-CATATGCTGTCGCACCTGCGCT-3’ | Generation of recombinant GtrS-LBR |
| GtrS-Lbd.r | 5’-GGATCCTCAGAGCACCTGCTGC-3’ | Generation of recombinant GtrS-LBR |
| OprB.f | 5’-CTGCAGAGACAGATGACGGCAGAGAG-3’ | EMSA, footprint, primer extension, fusion to *lacZ* |
| OprB.r | 5’-AGATCTCGGGTCAGCCTGCGC-3’ | EMSA, footprint, primer extension, fusion to *lacZ* |
| Glk.f | 5’-CTGCAGCGATATCGCCGACCA-3’ | EMSA, footprint, primer extension, fusion to *lacZ* |
| Glk.r | 5’-AGATCTTGCAGCTTTACCGAGAGCCT-3’ | EMSA, footprint, primer extension, fusion to *lacZ* |
| Edd/gap.f | 5’-CTGCAGTGCGGCGGGTGACT-3’ | EMSA, footprint, primer extension, fusion to *lacZ* |
| Edd/gap.r | 5’-AGATCTGTTGATGGCCAGGCGG-3’ | EMSA, footprint, primer extension, fusion to *lacZ* |
| ToxA.f | 5’-CTGCAGACTCACCCTTGAGGCCC-3’ | EMSA, fusion to *lacZ* |
| ToxA.r | 5’-AGATCTCAATGGGGTGTCAGGTGCAT-3’ | EMSA, fusion to *lacZ* |
| MnzV.f | 5’-CTGCAGCGGGAACCAGACCG-3’ | EMSA |
| MnzV.r | 5’-AGATCTCTACGTGTTCGTGCCGGAC-3’ | EMSA |
| OmpR.f | 5’-CTGCAGCTTCGGCCAGGGC-3’ | EMSA |
| PoprB Mut. F | 5’-AAAACCTTCTTGTTGTTATTCGGAGCAA-3’ | Generation of GltR mutant box |
| PoprB Mut. R | 5’-CATGTGGGTTCGCCGGCC-3’ | Generation of GltR mutant box |
| GltR mut. F | 5’-GCGATCCACAGCACAATCGGAG-3’ | Generation of GltR mutant box |
| GltR Mut. R | 5’-CTCCGATTGTGCTGTGGATCGC-3’ | Generation of GltR mutant box |

The mismatch codon is underlined.
